# Knowledge and Attitude Towards Antibiotic Usage: A Questionnaire-Based Survey Among Pre-Professional Students at King Saud bin Abdulaziz University for Health Sciences on Jeddah Campus, Saudi Arabia

**DOI:** 10.3390/pharmacy8010005

**Published:** 2020-01-06

**Authors:** Syed Faisal Zaidi, Rakan Alotaibi, Abdulaziz Nagro, Muath Alsalmi, Hidaya Almansouri, Muhammad Anwar Khan, Aslam Khan, Ismail Memon

**Affiliations:** 1 College of Medicine, King Saud bin Abdulaziz University for Health Sciences, Jeddah 21423, Saudi Arabia; 2 King Abdullah International Medical Research Center, Jeddah 21423, Saudi Arabia; 3 College of Science and Health Professions, King Saud bin Abdulaziz University for Health Sciences, Jeddah 21423, Saudi Arabia; 4 College of Science and Health Professions, King Saud bin Abdulaziz University for Health Sciences, Riyadh 11481, Saudi Arabia

**Keywords:** antibiotic knowledge, attitudes, usage, resistance, public awareness

## Abstract

**Introduction:** Knowledge and attitudes towards proper antibiotic usage among students in medical and other health allied fields play a vital role in limiting the pandemic of antibiotic resistance. This study aimed to assess knowledge and attitudes toward antibiotic usage among pre-professional students at King Saud bin Abdulaziz University for Health Sciences on Jeddah Campus, Saudi Arabia. **Methods:** A self-administered cross-sectional survey of 347 first year students was conducted at the College of Science and Health Professions, King Saud bin Abdulaziz University for Health Sciences using a validated questionnaire. **Results:** Nearly 63% of the students had a moderate level of knowledge. Two third of the students (69.7%) correctly knew that antibiotics are indicated for the treatment of bacterial infections. However, about 36% of the students incorrectly thought that antibiotics are also used to treat viral infections, while 28.5% were not sure. Only 38.8% of the students were aware of antibiotic resistance phenomena in relation to the overuse of antibiotics. Furthermore, only 27.3% correctly knew that penicillin is an antibiotic, and 74.4% were not sure if Paracetamol is an antibiotic. With regard to attitudes, 25.7% believed that taking antibiotics when having cold symptoms could help them to recover faster, while 39.6% expected antibiotics to be prescribed for common cold symptoms. **Conclusion:** The students have misconception regarding antibiotic use indications. Therefore, awareness campaigns are needed to promote student’s use of antibiotics in young generations particularly among the pre-professional health sciences students.

## 1. Introduction

The rise of antibiotic resistance is increasingly menacing to human health worldwide. In its 2007 report, the World Health Organization (WHO) referred to antibiotic resistance as one of the major concerns to human wellbeing in the 21st century [1]. On other side, the pharmaceutical industry is failing to keep up, through the development of new antibiotics, to counteract this great threat to global public health security [2]. Irrational use of some antibiotics without prescription, especially in developing countries, has contributed to the emergence of antibiotic resistance [3]. This trend has led to the ineffectiveness of many antibiotics to treat many infectious diseases that were once treatable, leading to a tremendous increase in mortality and morbidity [4].

One of the misuses contributing to the ineffectiveness of antibiotics in the era of resistance is the inappropriate prescribing of antibiotics for illnesses where there are insignificant clinical benefits to its use. An example of inappropriate prescribing is with the presentation of upper respiratory tract infections (URTIs) that are caused by viral infections, not bacterial infections [5,6]. Antibiotics are manufactured to destroy or inhibit the growth of bacteria only and cannot be used to eradicate viruses [6]. In addition, antibiotic overuse is a leading factor, among the many complex factors, of antibiotic resistance [7]. In Saudi Arabia between 2010 and 2015, antibiotics were the second most commonly used drugs [8]. The pattern of antibiotic consumption in one of the major hospitals in Saudi Arabia for admitted patients shows that around two thirds of the prescriptions were broad-spectrum antibiotics where one third of the cases were without documented diagnoses [9]. Additionally, easy accessibility to over-the-counter antibiotics without a valid medical prescription in Saudi Arabia coupled with the misconception of antibiotics usage is a main factor contributing to antibiotic resistance [10].

Moreover, a lack of appropriate knowledge on the behavior and attitude towards the usage of antibiotics has been suggested [11,12,13]. A report published by WHO in 2000 suggests three steps to eliminate the issue of antibiotic resistant. (1) Better access to medical services. (2) Reduction of unnecessary antibiotic use. (3) Not sharing medication with others [14]. Hence, there is a need to raise awareness among the whole community, particularly, among the students in medical and other health-allied fields, about the proper use of antibiotics in order to control antibiotic resistance and address these three main underlying issues.

Around the world, there is a lack of studies about the perceptions of healthcare students, although they play a vital role in raising awareness of health-related issues [15]. In addition, in Saudi Arabia only two studies have been conducted targeting medical and dental students regarding their knowledge, attitude, and perception of antibiotic prescribing guidelines [16,17]. Therefore, this study seeks to investigate the knowledge and attitudes among medical and other health-allied students at College of Science and Health Professions (COSHP) at King Saud bin Abdulaziz University for Health Sciences (KSAU-HS)–Jeddah campus to gain further understanding in order to implement more effective strategies in raising awareness.

## 2. Methodology

### 2.1. Study Design and Population

A validated self-administered questionnaire was used in a cross-sectional survey. It was conducted during the academic year of 2017–2018 among freshmen students attending COSHP at KSAU-HS, Jeddah campus. Arrangements were made with student affairs of COSHP in advance to distribute the survey under the supervision of the researchers. The study was approved by the Institutional Review Board of King Abdullah International Medical Research Center, Jeddah, Saudi Arabia.

The number of COSHP freshmen attendees was about 800 students. This number was used to calculate the required sample size for this study. The response distribution was assumed to be 50% and the confidence interval was established at 95% with a 5% margin of error. Sample size was computed by using the website Raosoft software and the minimum needed sample size for the survey was determined to be 260 accounting for a 15% non-response rate [18]. The carried calculation using the software was under the assumption that there would be more than 30 respondents and it was based on normal distribution [18]. Eventually, 347 students were included in this study. The only eligibility requirement for subjects to be included was that they were first year students at COSHP and were ready to participate in the study.

### 2.2. Development of Questionnaire

The questionnaire was adapted from a previous study and was modified to befit the population of this study [19]. The questionnaire consisted of four parts. Part 1 aimed to obtain the demographic characteristics of the students. Part 2 obtained information in regard to recent usage of antibiotics among students for the past six months. Students were asked to provide additional information concerning the source and reason for taking, if they had taken, antibiotics in the past six months. Part 3 of the questionnaire was comprised of 14 statements to assess the subjects’ knowledge of antibiotics. Statements evaluated the purpose of antibiotics (five statements), identification of antibiotics (four statements), peril of antibiotics (side effects, resistance, and allergic reaction: one statement for each), and consummation of treatment course (two statements). Students were asked to select one of three options provided: “Yes”, “No”, or “Not Sure”. Part 4 consisted of nine statements, and it was designed to evaluate students’ attitudes towards antibiotics, including: antibiotics usage during colds, students’ expectations of doctors, consummation of treatment course, sharing of antibiotic, retaining stocks of antibiotic for emergency use, leftover use of antibiotics, adhesion to antibiotic label instruction, reading the expiry date before taking antibiotics, and providing consultation to others during colds. The Likert-type scale was used to measure students’ attitudes ranging from “Strongly Agree” to “Strongly Disagree.” Responses with “Agree” and “Strongly Agree” were considered as having agreed while “Disagree” and “Strongly Disagree” as having disagreed for the purpose of analysis simplicity. Positive responses/attitudes would denote the suitability of using antibiotic whereas negative responses/attitudes would imply the inappropriateness of antibiotic usage. The options “Disagree” or “Strongly Disagree” for statements 1 to 6 and 9, and “Agree” or “Strongly Agree” for statements 7 and 8 indicated a positive response/attitude.

The questionnaire was prepared in two language versions, English and Arabic (the official language of Saudi Arabia). The questionnaire was initially developed in English and then translated to Arabic. Content and face validation were accomplished by senior faculty members in medical education (a pharmacologist and a statistician). Face validation of Arabic translation was done by senior local resident fluent in Arabic. The questionnaire was modified based on provided feedback and unanimous agreement by all members involved. A pilot study was carried out with 30 randomly selected students. Based on the pilot study, students were found to understand and complete the provided survey. Therefore, no further modification was needed preceding the actual survey. Reliability testing was performed in a previous study regarding attitude responses from the pilot study, and the results reflected internal consistency of areas tested with the Cronbach’s α value of 0.76 [19].

### 2.3. Data Analysis

In part 3 of the questionnaire where students’ level of knowledge of antibiotics was assessed, questions were marked in a dichotomous manner. One point was given for each correct response and zero for each incorrect or unsure answer with the maximum attainable score being 14. A scoring system (0–14) to evaluate the level of knowledge was used based on given responses. The aggregate score of knowledge was classified into three levels betokened by good (10–14), moderate (5–9), and poor (0–4). Data analysis was executed using IBM Statistic SPSS (SPSS Inc., Chicago, IL, USA) version 25.0. Descriptive analysis was employed to sum up the data set (demographic characteristics, recent use of antibiotics, and knowledge, and attitudes toward antibiotic usage). To test the influence of demographic characteristics on knowledge and attitude, Chi square or Fishers exact tests were applied wherever suitable, and *p*-values were obtained for each test. The level of statistical significance was set at *p* < 0.05.

## 3. Results

A total of 347 questionnaires were distributed to first year students attending COSHP at KSAU-HS, Jeddah campus. Twelve of the questionnaires were found incomplete and therefore were excluded from the analysis (useable rate of 97.14%). As illustrated in the summary of demographic characteristics in Table 1, the mean age of freshmen was 18.75 (SD = 0.606) and the majority of them were male (61.7%). Most of the respondents’ parents have achieved a college/university degree (72.9% of fathers and 66.6% of mothers) and 58.9% of their parents have an income above SR.15,000. From the results obtained regarding the usage of antibiotics (Table 2), almost half (45.5%) of the respondents reported using antibiotics in the last 6 months. Of those who took antibiotics, 25.3% obtained it without a prescription by using a left-over antibiotic (47.5%), using someone’s antibiotic (25%), or using other sources (27.5%). On the other hand, 74.7% obtained their antibiotics using a doctor prescription. The reasons for taking antibiotics were mostly due to fever, pain, and inflammation (77.8%), or respiratory illness (23.4%).

Most respondents (62.8%, n = 218) had a moderate level of knowledge (Table 3). Poor level of knowledge was found to be associated with the educational status, income of parents, and gender (Table 4). Over half, 52.9%, of respondents whose fathers had primary or lower educational status had a poor level of knowledge; similarly, 45.7% of respondents whose mothers had primary or lower educational status had a poor level of knowledge. In addition, 35.5% of respondents with parents’ having an income less than 5000 had a poor knowledge compared to less than 33.9% in other groups (*p* = 0.022). Finally, poor level of knowledge was found to be higher in males (31.3%) in comparison to females (21.1%; *p* = 0.042).

As for the knowledge of the role of antibiotics (Table 5), two third of the students could correctly identify that antibiotics are indicated for the treatment of bacterial infections which was the highest correct response (69.7%). However, only 35.7% of the students could correctly identify that antibiotics cannot be used to treat viral infections. It also was the only statement significantly associated with gender regarding the knowledge of the role of antibiotics (*p* = 0.015). The highest incorrect response in the knowledge domain was in identifying that antibiotics can cure all infections (72%). Regarding assessment of identification of antibiotics, only 4.9% of the students could correctly identify that diphenhydramine is not an antibiotic. Nonetheless, it is essential to point out that the highest students’ responses in this section were the unsure response in comparison to other sections in assessment of knowledge. Furthermore, 38.3% of the students could correctly identify that overuse of antibiotics could cause antibiotic resistance while 45.0% provided incorrect responses and 16.7% were unsure. It is important to note that this is the only statement in this section with significant correlation to both of parents’ educational status (father: *p* = 0.005; mother: *p* = 0.002). More than two thirds (70%) of the students incorrectly identified that antibiotics do cause side effects. As for the feedback concerning to the completion of treatment course of antibiotic even when symptoms are improving, more than half (54.5%) gave an incorrect response. However, a high percentage (71.2%) provided a correct response to the statement about the effectiveness of antibiotics is reduced if a full course of antibiotic is not completed. For all the knowledge statements evaluated, poor level knowledge was mostly found among students with parents with a primary or lower education level.

In general, students were found to have more negative attitudes towards antibiotics in almost all of the statements regarding attitudes (Table 6). The highest negative response was found in giving one’s own antibiotic to a sick family member (88.4%) followed by keeping leftover antibiotics to use later in case of a respiratory illness (78.9%). Similarly, 78.6% of respondents stated that they would recommend antibiotics to a sick family member if they ask their advice; which was significantly associated with the income of parents (*p* = 0.001). In addition, about 55% of the respondents stop taking antibiotics when they start feeling better. Regarding this statement, there is a significant association with the educational level of the father (*p* = 0.008) and income of parents (*p* = 0.035). Keeping antibiotics stocks at home in case of emergency was stated by 54.6% of the population of the study. Moreover, 85.2% of the respondents were unsure if they take antibiotics according to the instruction on the label and 80.6% were not sure if they look at the expiry date of antibiotics.

## 4. Discussion

Knowledge and attitudes towards proper antibiotic usage, among common people, in general, and among health allied field workers, in particular, play a vital role in limiting antibiotic resistance. In this study we have assessed the knowledge and attitudes of pre-professional students toward antibiotic usage. Regarding the knowledge level, the findings of the study showed that only 35.7% of pre-professional first year students could identify that antibiotics are not useful in treating viral infections, whereas 35.2% thought otherwise and the remaining were not sure. This proportion is comparable to a study done in Oceania (34.4%, 95% CI 66.5–87.6), but less than a systemic review and meta-analysis of 24 studies (53.9%, 95% CI 41.6–66.0) [20].The possible reason for this lack of knowledge regarding this statement is the use of the general term “inflammation” by healthcare providers in Saudi Arabia instead of specifying the causative agent of the infection; bacterial or viral. In addition, this statement was the only statement significantly associated with gender regarding the knowledge of the role of antibiotics (*p* = 0.015).

The findings of this study pointed out that there is a lack of knowledge regarding the identification of antibiotics and other commonly used medications. Only 27.3% correctly identified that penicillin is an antibiotic and it was significantly associated with the educational level of the father. Meanwhile, 74.4% were not sure whether Paracetamol is an antibiotic or not and 57.6% were unsure when it came to aspirin. This proportion is less than a study done in Jordon where participants misused antibiotics for analgesics (28.1%) [20]. Such a lack of knowledge could be explained by several factors. One of which is that the public uses only the brand names when dealing with medications. In addition, it could be caused by not writing down the names of one’s medication or from a lack of clarification by healthcare providers; all of which can be backed up by more investigation in this area. 

More than half of the respondents (54.5%) had an incorrect knowledge regarding the need to complete a full course of antibiotics when symptoms are improving. Comparatively, a similar proportion was found in the systemic review and meta-analysis of 24 studies (47.1%) regarding this statement [20]. Correspondingly, about 55% of the respondents stop taking antibiotics when they start feeling better, however, 71.2% of the respondents correctly agreed that the effectiveness of treatment is reduced if a full course of antibiotic is not completed. This suggests that there is a lack of knowledge regarding the need and importance of complying with antibiotics medication.

Regarding the attitudes towards antibiotics, the study showed that students were found to have more negative than positive responses. The highest negative response was found in giving one’s own antibiotic to a sick family member (88.4%). This proportion is considerably higher compared to other studies done in Singapore (6.8%) and the Philippines (37%) [21,22]. This is followed by keeping leftover antibiotics to use later in case of a respiratory illness (78.9%). Similarly, 78.6% of respondents stated that they would recommend antibiotics to a sick family member if they ask their advice. The aforementioned negative attitudes could be due to the inappropriate prescriptions of antibiotics from primary care physicians. A study showed around 50% of antibiotic prescriptions in primary care settings are “inappropriate” and around 75% of total antibiotics prescriptions are for common diseases such as respiratory illness [20]. In addition, about 55% of the respondents stop taking antibiotics when they start feeling better, consequently, about the same proportion keep them in stocks at home to use in case of an emergency. This is comparable with the systemic review study where 47.1% of the respondents stop taking antibiotics when they start feeling better [20].

In this study, 64.2% of the students agreed that they will take antibiotics to relieve cold symptoms more quickly, which is considerably higher compared to the Riyadh, Saudi Arabia community where 31.1% of the respondents use antibiotics to treat the common cold [23]. Similarly, in comparison to other countries, the percentage was found to be higher than the general population of Malaysia (38%), South Africa (42%), and US (27%) [11,19,24]. However, in a meta-analysis pertaining 24 papers, 57.4% (95% CI 34.1–79.1) take antibiotics to prevent cold symptoms from getting worse, which is comparable to the proportion found in this study [20,23]. One reason for such a misconception is the irrational prescriptions of antibiotics for URTIs, which is mostly self-limiting and of a viral cause. As a result, it is conceived by the public that antibiotics are effective in treating such illnesses. Thus, patients with common cold symptoms would have high expectations of being prescribed with an antibiotic as a treatment [20]. In this study 36.7% of the respondents expect to have an antimicrobial prescription upon acquiring the common cold symptoms. This finding is considerably lower than the results found in the general population of Malaysia and the United States (47.3% and 48% respectively) [19,24]. In addition, this percentage (36.7%) regarding the expectation to have an antimicrobial prescription upon acquiring the common cold symptoms is considerably lower than the percentage of students who agreed that they will take antibiotics to relieve cold symptoms more quickly (64.2%). The possible reason for this is the lack of knowledge and misconception about the role of antibiotics. The 64.2% might have agreed to take antibiotics with common cold symptoms only because they believe it helps them get better more quickly.

Patients’ expectations and satisfaction play a vital role in unnecessary antibiotic prescription as suggested by previous studies [25,26,27]. In some countries, the use of value-based purchasing, where physicians’ payments as well as hospitals’ quality are evaluated based upon certain performance measures, one of which being patient satisfaction, is one determinant of antibiotic prescription [27]. Under such pressured clinical settings, overestimation and inaccurate measurement of expectation and satisfaction lead to unnecessary antibiotic prescription to meet such standards [26]. In addition, the patient–doctor relationship has a great impact to whether or not antibiotic is prescribed [25]. Physicians irrationally prescribe antibiotic to satisfy patients’ expectation even though patients realize that prescription of antibiotic is not needed [26]. Nonetheless, patients were more satisfied with a thorough explanation of their illness and its effects even without medications prescription [25,26,27]. Patients’ satisfaction is met with more thoughtfulness of their main complaint even without antibiotic prescription as mentioned in several studies [25,26,27].

Another factor that plays a role in the misuse of antibiotics and consequently antibiotic resistance is the inadequate supervision of antibiotics. According to a meta-analysis, globally, 78% of antibiotic requests from commercial pharmacies led to the supply of antibiotics [28]. Saudi Arabia (85%, 95% CI 72–99) along with Indonesia (91%, 95% CI 86–94), Ethiopia (85% 95% CI 75–92), and Syria (87%, 95% CI 82–91) are among the countries with the largest supply of non-prescription antibiotics [28]. However, it is noteworthy that as of 18 April 2018, the Saudi authorities implemented a strict law against selling of antibiotics without a prescription, as they are usually obtained from retail pharmacies, with consequences including license suspension, 6 months imprisonment, and 100,000 SAR fine [29]. In this study, which was conducted before the introduction of such laws, the percentage of non-prescription antibiotics was found to be significantly lower (25.3%) which could be contributed to the fact that the population of this study is pre-professional students. Further investigation regarding the effectiveness of introducing such laws in reducing non-prescription antibiotics supply and consequently decreasing antibiotics overuse is of a great importance.

The public misuse of antibiotics is not the only determining factor for this issue as healthcare providers should be held accountable as well. Unnecessary prescriptions and easy accessibility to over-the-counter antibiotics, such as generic types of penicillin and cephalosporin, in Saudi Arabia in the past few years are factors contributing to this issue [28]. This could be the result of several factors including, but not limited to, inaccurate overestimation of patients’ satisfaction for commercial benefit and high patients demands to get antibiotics. This could jeopardize safety of patients and their overall wellbeing leading to higher antibiotic resistance coupled with incapability of healthcare professionals to meet patients’ needs. Weighing the risk of the community in general and patients in particular versus the benefit of profit driven healthcare system is indispensable for this intricate issue.

Recently, the Ministry of Health in Saudi Arabia marked antibiotic awareness week in which it aims to shed light and alert the public on the issue of antibiotic misuse [30]. The campaign was held at King Fahd General Hospital in Jeddah, and its main purpose was to boost the awareness and urge caution when using antibiotics. It also illuminated the fact that Saudi Arabia has a dangerously high antibiotic consumption while there is in fact a shortage of antibiotics in other countries. This easy access to antibiotics may also be propelling unnecessary use in the Kingdom and thus leading to the issue of resistance [31]. Even though this is a positive initiative towards solving the issue of antibiotic misuse and resistance, it is not only needed within hospitals but to a wider-reaching audience, such as in malls and public schools. This could result in better knowledge and attitudes towards this issue as the lack of knowledge and higher negative attitude indicated in this study show a clear need for a proposed solution.

This study should take into account the limitations that may affect the interpretation of the findings and thus impact upon its conclusion. The study was conducted on pre-professional first year students in a college that only accepts the top students among high-school graduates and gender ratio, even though efforts were made to obtain matched gender ratio, in the study sample indicates over representation of male students denoting selection bias. Thus, generalizing the results to a large scale may lack validity. Additionally, this is a self-report study and the analyzed results were highly dependent upon students’ recalling ability and honesty. Another limitation is the nature of the study-cross sectional—which reflects students’ knowledge and attitude of antibiotic at one point in time. A change in knowledge and attitude may need to be assessed by the end of their second year—in which they would have taken a pharmacology class.

## 5. Conclusions

The students have misconceptions regarding antibiotic use indications, which might be a factor in unnecessary prescription. Better communication between physicians and patients toward understanding the difference between viral and bacterial infections may increase the knowledge as well as the positive attitude regarding antibiotics and therefore lead to decreased patient demand and appropriate prescription. The findings in this study are fundamental for future interventional directions to lessen the gap in knowledge and attitude in young generation, particularly among the pre-professional health sciences students. Awareness campaigns are needed to convey specific messages and promote prudent use of antibiotics. Another possible intervention is the governmental use of social media as a platform to deliver appropriate knowledge about antibiotic as well as the negative effects of its overuse i.e., antibiotic resistance to a larger audience.

## Figures and Tables

**Table 1:** Summary of demographic characteristics.

Characteristics	Number	Percentage (%)
***Age* (332)**		
18.75 ± 0.606	332	95.6
***Gender* (347)**		
Male	214	61.7
Female	133	38.3
***Highest educational status of father* (346)**		
Primary or lower	30	4.9
Secondary	80	21.9
College/University	231	72.9
***Highest educational status of mother* (341)**		
Primary or lower	13	8.6
Secondary	112	23.1
College/University	209	66.6
***Monthly income* (329)**		
<5000 SAR	13	3.7
5000–15,000 SAR	112	32.3
>15,000 SAR	209	60.2

**Table 2:** Usage of antibiotics.

Recent Use (within 6 Months)	Number (n = 347)	Percentage (%)
Yes	158	45.5
No	189	54.5
***Source of antibiotic***		
Prescribed	118	74.7
Without prescription Leftover antibiotic Used someone’s antibiotic Others	40	25.3
	19	47.5
	10	25.0
	11	27.5
***Reasons for taking antibiotic***		
Fever/Pain/Inflammation	123	77.8
Respiratory illness	37	23.4
Urinary tract infection	5	3.1
Skin problem/wound	12	7.5
Others	12	7.5

**Table 3:** Level of knowledge.

Level of Knowledge	Total Score	n (%)
Poor	0–4	95 (27.4)
Moderate	5–9	218 (62.8)
Good	10–14	34 (9.8)

**Table 4:** Association of demographic characteristics with level of knowledge.

Characteristics	Level of Knowledge			*p* Value (X^2^ Test/Fisher Exact Test)
	Poor (0–4)	Moderate (5–9)	Good (10–14)	
***Gender***				
Male	67 (31.1%)	131 (61.2%)	16 (7.5%)	**0.042**
Female	28 (21.1%)	87 (65.9%)	18 (13.5%)	
***Educational status of father***				
Primary or lower	9 (52.9%)	7 (41.2%)	1 (5.9%)	0.022 *
Secondary	25 (32.9%)	48 (63.2%)	3 (3.9%)	
College/University	60 (23.7%)	163 (64.4%)	30 (11.9%)	
***Educational status of mother***				
Primary or lower	16 (45.7%)	14 (40.0%)	5 (14.3%)	0.015 *
Secondary	22 (27.5%)	53 (66.3%)	5 (6.3%)	
College/University	56 (24.2%)	151 (65.4%)	24 (10.4%)	
***Monthly income***				
<5000 SAR	3 (35.5%)	4 (50%)	1 (12.5%)	0.2 *
5000–15,000 SAR	38 (33.9%)	61 (54.5%)	13 (11.6%)	
>15,000 SAR	46 (22.5%)	139 (68.1%)	19 (9.3%)	

The level of statistical significance was set at *p* < 0.05; *: Fisher exact test; Bold font correspond to significant values.

**Table 5:** Association of demographic characteristics with knowledge statements.

Statement	Correct Answer	Incorrect Answer	Unsure	*p* Value (X^2^ Test/Fisher Exact Test)			
				Gender	Education of Father	Education of Mother	Income
***Role of Antibiotic***							
Antibiotics are medicines that can kill bacteria.	242 (69.7%)	33 (9.5%)	72 (20.8%)	0.188	0.430 *	0.096 *	0.203 *
Antibiotics can be used to treat viral infections.	124 (35.7%)	122 (35.2%)	101 (29.1%)	**0.015**	0.585	0.929 *	0.963 *
Antibiotics can cure all infections.	9 (2.6%)	250 (72.0%)	88 (25.4%)	0.643	0.287 *	0.583 *	0.629
Antibiotics are indicated to relieve pain/inflammation.	209 (60.2%)	79 (22.8%)	59 (17.1%)	0.587	0.651 *	0.389 *	0.192 *
Antibiotics are used to stop fever.	140 (40.3%)	113 (32.6%)	94 (28.1%)	0.079	0.701	0.384 *	0.768 *
***Identification of Antibiotic***							
Penicillin is an antibiotic.	93 (26.8%)	72 (20.7%)	176 (51.5%)	0.883	0.005 *	0.589 *	0.230 *
Aspirin is a new generation of antibiotic.	44 (12.7%)	103 (29.7%)	198 (57.6%)	**0.004**	0.009 *	0.404 *	0.399 *
Paracetamol is considered as an antibiotic.	29 (8.4%)	58 (16.7%)	260 (74.9%)	**0.020**	0.05 *	0.586 *	0.972 *
Diphenhydramine is not an antibiotic.	17 (4.9%)	15 (4.3%)	315 (90.8%)	0.352	0.047 *	0.781 *	0.773 *
***Dangers of Antibiotic***							
Overuse of antibiotics can cause antibiotic resistance.	133 (38.3%)	156 (45.0%)	58 (16.7%)	0.792	0.005 *	0.002 *	0.077 *
Antibiotics may cause allergic reaction.	189 (54.5%)	18 (5.2%)	140 (40.3%)	0.356	0.102 *	0.227*	0.160 *
All antibiotics do not cause side effects.	18 (5.2%)	243 (70.0%)	86 (24.8%)	0.398	0.312 *	0.097*	0.573 *
***Completion of Treatment Course***							
You can stop taking a full course of antibiotic if your symptoms are improving.	118 (34.0%)	189 (54.5%)	40 (11.5%)	0.559	0.526	0.213 *	0.128 *
The effectiveness of treatment is reduced if a full course of antibiotic is not completed.	247 (71.2%)	36 (10.4%)	64 (18.4%)	0.137	0.714 *	0.420 *	0.441 *

The level of statistical significance was set at *p* < 0.05; *: Fisher exact test; Bold font correspond to significant values.

**Table 6:** Association of demographic characteristics with attitude statements.

Statement	Agree	Disagree	Unsure	*p* Value (X^2^ Test/Fisher Exact Test)			
				Gender	Education of Father	Education of Mother	Income
When I get a cold, I will take antibiotics to help me get better more quickly.	222 (64.2%)	35 (10.1%)	89 (25.7%)	0.297	0.264	0.164 *	0.078 *
I expect antibiotics to be prescribed by my doctor if I suffer from common cold symptoms	127 (36.7%)	82 (23.7%)	137 (39.6%)	0.694	0.477	0.578 *	0.453 *
I normally stop taking antibiotics when I start feeling better.	189 (54.8%)	25 (7.2%)	131 (38.0%)	0.570	**0.008**	0.116 *	0.035 *
If my family member is sick, I usually will give my antibiotics to them.	306 (88.4%)	19 (5.5%)	21 (6.1%)	0.562	0.208 *	0.639 *	0.783 *
I normally keep antibiotics stocks at home in case of emergency.	189 (54.6%)	36 (10.4%)	121 (35.0%)	0.054	0.402	0.918 *	0.857 *
I will use leftover antibiotics for a respiratory illness.	273 (78.9%)	58 (16.8%)	15 (4.3%)	0.589	0.731 *	0.714 *	0.906 *
I will take antibiotics according to the instruction on the label. *	21 (6.1%)	30 (8.7%)	293 (85.2%)	0.557	0.220 *	0.227 *	0.394 *
I normally will look at the expiry date of antibiotics before taking it *.	34 (9.9%)	33 (9.6%)	278 (80.6%)	0.978	0.525 *	0.209 *	0.828 *
When a family member or a friend feels sick, I recommend antibiotics.	272 (78.6%)	46 (13.3%)	28 (8.1%)	0.709	0.483 *	0.174 *	**0.001**

The level of statistical significance was set at *p* < 0.05; *: Fisher Exact test; Bold font correspond to significant values.
